# Expression of LacdiNAc Groups on N-Glycans among Human Tumors Is Complex

**DOI:** 10.1155/2014/981627

**Published:** 2014-05-18

**Authors:** Kiyoko Hirano, Akio Matsuda, Takashi Shirai, Kiyoshi Furukawa

**Affiliations:** ^1^Laboratory of Glyco-Bioengineering, The Noguchi Institute, Itabashi, Tokyo 173-0003, Japan; ^2^Laboratory of Glycobiology, Department of Bioengineering, Nagaoka University of Technology, Nagaoka, Niigata 940-2188, Japan

## Abstract

Aberrant glycosylation of proteins and lipids is one of the characteristic features of malignantly transformed cells. The GalNAc**β**1 → 4GlcNAc (LacdiNAc or LDN) group at the nonreducing termini of both N- and O-glycans is not generally found in mammalian cells. We previously showed that the expression level of the LacdiNAc group in N-glycans decreases dramatically during the progression of human breast cancer. In contrast, the enhanced expression of the LacdiNAc group has been shown to be associated with the progression of human prostate, ovarian, and pancreatic cancers. Therefore, the expression of the disaccharide group appears to be dependent on types of tumors. The mechanism of formation of the LacdiNAc group in human tumors and cancer cells has been studied, and two **β**4-N-acetylgalacto-saminyltransferases (**β**4GalNAcTs), **β**4GalNAcT3 and **β**4GalNAcT4, have been shown to be involved in the biosynthesis of this disaccharide group in a tissue-dependent manner. Transfection of the **β**4GalNAcT3 gene brought about significant changes in the malignant phenotypes of human neuroblastoma, indicating that this disaccharide group is important for suppressing the tumor growth.

## 1. Introduction

The nonreducing terminal GalNAc*β*1 → 4GlcNAc (LacdiNAc or LDN) group is found in N- and O-glycans of both vertebrate and invertebrate glycoproteins. The disaccharide group is highly expressed in parasitic helminthes such as* Schistosoma mansoni* [1]. However, the LacdiNAc group is a rare structure in mammalian glycoproteins, and this is because only a limited number of glycoproteins such as bovine pituitary glycohormones have been shown to possess this novel disaccharide structure (reviewed in [2, 3]). However, recent progress in analytical techniques for glycan structures has shown that the LacdiNAc group is expressed in many mammalian glycoproteins though in a very small amount [4]. The LacdiNAc group is mainly found in N-glycans and occasionally modified by sulfation [5–7], sialylation [8–11], and/or fucosylation [8, 11]. A few studies also showed that the disaccharide group is expressed on O-glycans attached to bovine proopiomelanocortin and murine zona pellucida glycoprotein 3, and it is modified further [12, 13]. To date, the disaccharide group with or without modification has been shown to play important roles in the regulation of half-life of circulating pituitary glycohormone, lutropin, in blood [14–16] and in the functional differentiation of bovine mammary epithelial cells [17, 18].

Many years ago, we showed that the expression level of the LacdiNAc group decreases in N-glycans of human breast cancers according to the malignant stages and relates to survival rates of the patients [19]. However, in the case of human prostate, ovarian, and pancreatic cancers, the expression of the LacdiNAc group is enhanced [20–23], indicating that the up- or downregulation of the disaccharide expression appears to be dependent on types of the tumors. Here, in this paper, we summarized the relationship of this disaccharide expression with tumor malignancy and its biosynthesis in human tumors.

## 2. Transformation-Associated Changes in Expression of LacdiNAc Group

Alteration of structures of glycans attached to proteins and lipids upon malignant transformation of cells is well documented [24, 25]. A large number of tumor-associated carbohydrate antigens such as Lewis X, sialyl Lewis X, Tn, and Globo H groups have been well characterized and used as tumor markers in respective human cancers [26]. To detect more specific diagnostic markers for individual cancers, the glycomic analysis is now being conducted.

**Figure 1:**
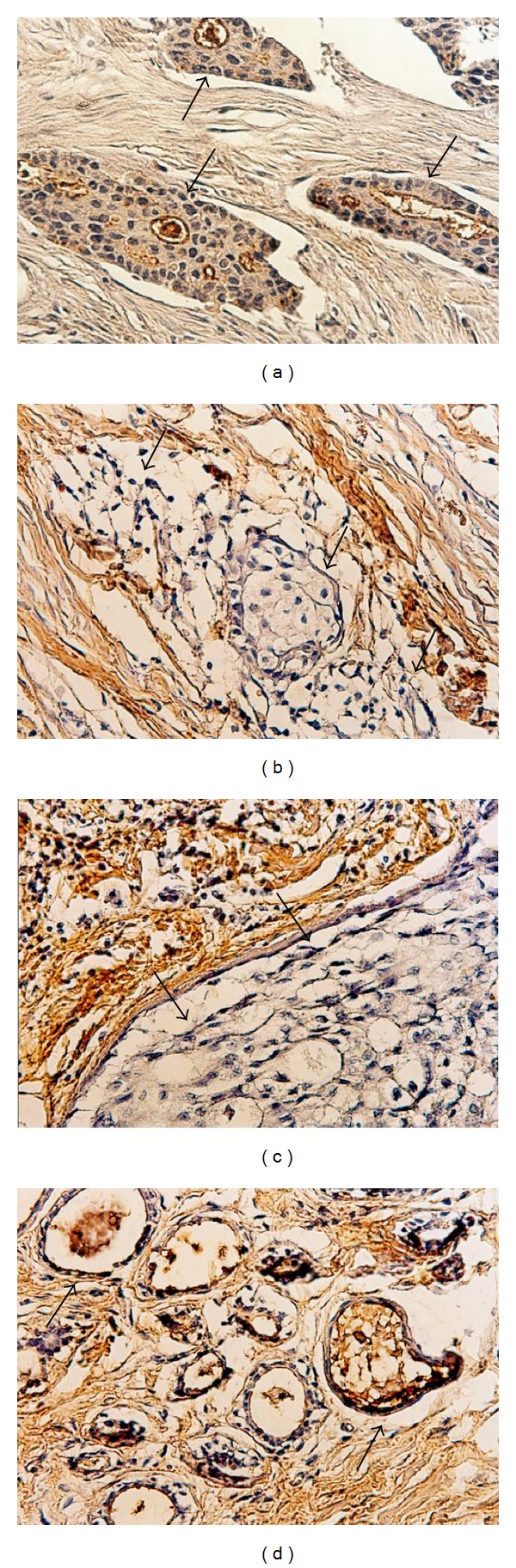
Histochemical staining with* Wisteria floribunda* agglutinin (WFA) of tissue sections from breast cancer patients. Panels (a), (b), and (c) indicate the tissues from patients with stages I, II, and III, respectively, and panel (d) indicates one of patients with fibrocystic disease of the breast. Arrows indicate carcinoma lesions. WFA binds glycans with the LacdiNAc group described in [19]. The WFA-positive staining shows brown color.

We initially found the LacdiNAc structure in the N-glycans of CD36 isolated from bovine milk fat globule membranes and then in those of many bovine mammary epithelial membrane glycoproteins [9, 27]. Quite interestingly, the expression of the LacdiNAc group in N-glycans was associated with functional differentiation of bovine mammary epithelial cells [18]. We then asked a question whether the expression of the LacdiNAc structure is affected by the malignant transformation of the mammary epithelial cells. However, no transformed bovine mammary epithelial cells were available at that time and are not even now, and, consequently, we examined the glycan expression levels using human breast cancer specimens. The results showed that the expression level of the LacdiNAc group decreases in primary carcinoma lesions when compared with samples from the surrounding normal tissues and that its reduced expression levels in primary carcinoma lesions correlate with advancing clinical stages (Figure 1) and prognostic status [19]. These results indicate that the LacdiNAc group is a differentiation marker and could be a tumor marker for human breast cancer. To investigate biological significance of the LacdiNAc group in the N-glycans in human breast cancers, we transfected the cDNA of *β*4-N-acetylgalactosaminyltransferase 4 (*β*4GalNAcT4), which contributes to the biosynthesis of the LacdiNAc group, into human breast cancer cell line, MDA- MB-231, and examined its growth activity* in vivo*. The preliminary results indicate that the tumor growth is strongly inhibited by the expression of the LacdiNAc group in the N-glycans (Hirano et al., manuscript in preparation). This suggests that the expression of the LacdiNAc group in N-glycans can suppress the tumor growth. The similar results were obtained from human neuroblastoma cells which showed reduced migratory and invasive activities when transfected with the *β*4GalNAcT3 cDNA [28]. Therefore, it is quite important to elucidate the molecular mechanism of how the LacdiNAc groups expressed in glycoproteins function to suppress the malignant properties of the tumors.

**Figure 2:**
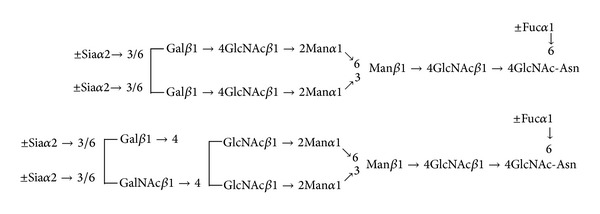
Structures of N-glycans of PSA. PSA samples were purified from sera of patients with prostate cancers and from patients with BPH. Structures of N-glycans were determined by MALDI-QIT TOFMS. Fuc: fucose; Gal: galactose; GalNAc: N-acetylgalactosamine; GlcNAc: N-acetylglucosamine; Man: mannose; and Sia: N-acetylneuraminic acid.

In contast to human breast cancers and neuroblastomas, enhanced expression of the LacdiNAc group on N-glycans of prostate specific antigen (PSA) purified from sera of patients with prostate cancer has been observed. PSA, which is widely used as a diagnostic marker for human prostate cancers, possesses one potential N-glycosylation site, and its glycan structure has been shown to change during the tumor progression [29–31]. The LacdiNAc group has been found in the N-glycans of PSA purified from seminal fluids of healthy individuals [32, 33] and from a human prostate cancer cell line, LNCaP, and the amounts of the LacdiNAc group were shown to increase in PSA from the prostate cancer cells [33]. We analyzed the structures of N-glycans attached to PSA purified from sera of patients with prostate cancers and of those with benign prostatic hyperplasia (BPH) [20]. As shown in Figure 2, PSA from patients with BPH contained mainly biantennary complex-type glycans with or without the core-fucosylation and/or sialylation, whereas PSA from patients with prostate cancer contained increased amounts of similar biantennary complex-type glycans but with the one LacdiNAc group which is partly sialylated. Quite interestingly, PSA from the LNCaP cells only contained neutral N-glycans [33]. However, we found that PSA from other prostate cancer cell lines, MDA PCa 2b, possesses N-glycans with the sialylated LacdiNAc group found in PSA from sera of patients with prostate cancer (Hirano et al., unpublished data). Other investigators reported the increased binding of PSA from sera of patients with prostate cancer but not with BPH to TJA-II (*Trichosanthes japonica *agglutinin-II), which interacts with glycans terminated with the GalNAc*β*1 →  group in addition to those with the *α*-1,2-linked fucose residue [34], suggesting that the expression level of the LacdiNAc group on the PSA glycans increases in human prostate cancer.The ratios of TJA-II-bound PSA to total PSA in sera of prostate cancer patients and in those of BPH patients were reported to be 8.3 ± 5.6% and 1.0 ± 0.55%, respectively [21]. Therefore, the different binding ratios of PSA samples toward TJA-II could be useful for judging patients with prostate cancer from those with BPH [21].

Likely in human prostate cancers, the increased expression of the LacdiNAc group has been observed for N-glycans of ribonuclease I isolated from a human pancreatic cancer cell line, Capan-1, while no such glycans were detected in the ribonuclease I from healthy individuals [23]. It has been also reported that not only exogenously expressed erythropoietin but also endogenous cellular glycoproteins synthesized in a human ovarian cancer cell line, SKOV3, contain N-glycans bearing the LacdiNAc groups [22]. Therefore, the LacdiNAc group may serve as a potential diagnostic marker for human pancreatic cancer and ovarian cancer. The reason why the expression of the LacdiNAc group is differentially regulated among human cancers remains to be elucidated.

## 3. Formation of LacdiNAc Group

It is of interest to know how the formation of the LacdiNAc group is regulated in tumors and cancer cells. To date, two human *β*4GalNAcTs, *β*4GalNAcT3 (**β*4GalNAcT3*, GeneBank AB089940) and *β*4GalNAcT4 (**β*4GalNAcT4,* GeneBank AB089939), were isolated, and they showed about 43% homology at the amino acid level [35, 36]. The *β*4GalNAcT3 and *β*4GalNAcT4 were shown to belong to the human *β*4-galactosyltransfease family and to be highly homologous to chondroitin sulfate synthases [35, 36]. These enzymes transfer N-acetylgalactosamine (GalNAc) from UDP-GalNAc to nonreduced terminal N-acetylglucosamine (GlcNAc) of N- and O-glycans in a *β*-1,4-linkage. Although both enzymes exhibit the same substrate specificities* in vitro*, they show different tissue distribution. The *β*4GalNAcT3 gene is expressed abundantly in human stomach, colon, and testis, and the *β*4GalNAcT4 gene is expressed in human ovary and brain [36]. Immunohistochemical analysis showed that the expression of *β*4GalNAcT3 and the LacdiNAc group is limited to the supernuclear region of the surface mucous cells in human gastric mucosa [37].

Several lines of studies have shown that the expression of the *β*4GalNAcTs is closely associated with the tumor formation. In human colon cancer, the transcript levels of *β*4GalNAcT3 were upregulated compared to that of the normal counterparts [38]. When the *β*4GalNAcT3 gene was overexpressed in a human colon cancer cell line, HCT116, the potentials of cell adhesion to extracellular matrix, migration, colony formation, and invasion of the cells increased significantly. Furthermore, the enhanced *β*4GalNAcT3 gene expression promoted tumor growth and metastasis of the HCT116 cells in nude mice. Biochemical analysis of this phenomena showed that the expression of the *β*4GalNAcT3 gene results in the activation of focal adhesion kinase and of extracellular signal-regulated kinases, indicating that this enzyme plays a critical role in promoting malignant behaviors of human colon cancer through the integrin-mediated signaling pathway [38]. In the case of the prostate cancers, the upregulation of the transcripts of *β*4GalNAcT4 but not that of *β*4GalNAcT3 has been reported. This was also observed in the prostate cancer-derived cells [21]. These results are quite consistent with the increased expression of the LacdiNAc group on the N-glycans of prostate cancer-derived PSA [20, 21].

In contrast to the colon and prostate cancers described above, the expression of the *β*4GalNAcT3 gene appeared to suppress malignant properties such as cell proliferation, migration, and invasion of human neuroblastoma cells [28]. In fact, the immunohistochemical study showed that the expression of the *β*4GalNAcT3 gene is detected significantly in differentiated human neuroblastomas but not in the undifferentiated neuroblastic cells [28]. The increased expression of the *β*4GalNAcT3 in human neuroblastic tumors was well associated with a favorable histologic profile and an early clinical stage and showed a favorable prognosis for survival. Moreover, when the *β*4GalNAcT3 gene was overexpressed in neuroblastoma cell lines, SK-N-SH and SH-SY5Y, their proliferation, colony formation, migration, and invasion were decreased compared to those of mock-transfected cell. The high expression of the *β*4GalNAcT3 gene resulted in an increase of the expression level of the LacdiNAc group on the *β*1-integrin N-glycans, which lead to the decreased phosphorylation of FAK, Src, paxillin, Akt, and ERK1/2 proteins. Therefore, the *β*4GalNAcT3 gene plays an important role in suppressing the malignant properties of the neuroblastoma cells via the integrin-mediated signaling pathway [28].

Thus, it is clear that the decreased or increased expression of the LacdiNAc group on N-glycans is closely associated with human tumor progression (Table 1). However, there are many issues to be solved such as detailed mechanisms of the biosynthesis of the LacdiNAc group and its functions in the tumors before elucidating its differential effects on tumor growth. Which *β*4GalNAcT is responsible for regulating the malignant properties of the tumors and cancer cells? Do *β*4GalNAcT3 and *β*4GalNAcT4 have any distinct biological functions in particular tumors and cancer cells? The accumulation of the *β*4GalNAcT3 and *β*4GalNAcT4 transcripts was observed in human colon and prostate cancers [21, 38]. On the other hand, the expression of the *β*4GalNAcT3 gene in human neuroblastoma showed a contradictory result as described above [28]. Therefore, the reports so far described indicate that the expression of *β*4GalNAcT3 and *β*4GalNAcT4 and that of the LacdiNAc groups are differentially regulated in the individual tumors. It is quite interesting that high transcript level of the *β*4GalNAcT4 gene but not the *β*4GalNAcT3 gene is detected in human fetal tissues, particularly the brain, lung, and kidney, suggesting that the *β*4GalNAcT4 gene is important for normal growth and differentiation of these organs [36].

Are there any other glycosyltransferases responsible for producing the LacdiNAc group? Baenziger and his colleagues showed that the particular tripeptide motif, Pro-Xaa-Arg/Lys, on the acceptor glycoprotein molecules is required for the formation of the LacdiNAc group on N-glycans of pituitary glycohormone by *β*4GalNAcT [39, 40]. Later, they also showed that the carboxyl-terminal 19-amino acid residue of bovine carbonic anhydrase-VI, which contains N-glycans with the LacdiNAc group, is also crucial for the peptide specific recognition by *β*4GalNAcT. When *β*4GalNAcT3 and *β*4GalNAcT4 were expressed in CHO cells, these enzymes could efficiently transfer GalNAc to nonreducing terminal GlcNAc residues of N-glycans of chimeric glycoproteins containing these peptide motifs [41]. Moreover, their recent studies have demonstrated that both *β*4GalNAcT3 and *β*4GalNAcT4 are able to recognize the same peptide motifs as above to selectively add GalNAc to the *β*1,6-linked GlcNAc residue of core 2 O-glycans* in vitro* [42]. Interestingly, *β*4GalNAcT3 and *β*4GalNAcT4 exhibited similar but distinct specificities toward acceptor glycoproteins [42, 43]. However, many glycoproteins do not always contain these peptide motifs recognized by *β*4GalNAcTs but possess N-glycans with the LacdiNAc group [9–11, 44, 45]. These reports suggest the presence of another *β*4GalNAcTs that can produce the LacdiNAc group on the glycans. Indeed, bovine milk *β*4-galactosyltransferase I can transfer GalNAc to nonreducing terminal GlcNAc residues of N-glycans in the presence of *α*-lactalbumin [46], though the enzyme preferentially catalyzes the transfer of galactose to the nonreducing terminal GlcNAc residues in the nonlactating tissues. However, the LacdiNAc group is found not only in the mammary gland but also in many other organs and tissues, and, therefore, it might not be a case for *β*4-galactosyltransferase I to be involved in the LacdiNAc formation. In support of this, the expression of the LacdiNAc group on N-glycans was unchanged in the *β*4-galactosyltransferase I knockout mice [47].

It should be also noted that the sialylated form of the LacdiNAc group is present in the prostate cancer cells [20, 21], although the function of this modified LacdiNAc group remains to be elucidated. The sulfated form and sialylated form of the LacdiNAc group are recognized by the mannose receptor [14–16] and by the asialoglycoprotein receptor [48, 49], respectively. Therefore, the sialylated LacdiNAc group in the prostate cancer cells may have some roles for the tumor formation by promoting cell adhesion. The LacdiNAc group has been found on N-glycans but not on O-glycans of particular glycoproteins in human tumors and cancer cells. Probably the limited amounts of the glycoproteins expressed make it difficult to detect the LacdiNAc group on O-glycans. Indeed, both *β*4GalNAcT3 and *β*4GalNAcT4 can transfer GalNAc to *β*-1,6-linked GlcNAc residue of core 2 O-glycans [42] and of several mucin-type glycoproteins [12, 13], suggesting the presence of the LacdiNAc group on O-glycans.

Recently, the phosphorylated form of the LacdiNAc group, [GalNAc*β*1 → 4(PO_3_ → GlcNAc*β*1 → 6)], has been found in O-glycans of recombinant and endogenous human and bovine extracellular matrix/matrix-related proteins such as zona pellucida glycoprotein 3 and *α*-dystroglycans [50]. Since O-glycans of murine zona pellucida glycoprotein 3 are involved in the initial binding of the sperms to the eggs [13], the LacdiNAc group with or without phosphorylation on the O-glycans may have some critical roles in the fertilization. It is possible that the LacdiNAc group expressed on O-glycans of human tumors may have a novel function for tumor formation, too. The detailed functional mechanisms of the LacdiNAc group together with its modified groups have to be elucidated by future studies.

## 4. Conclusion

An increasing number of studies have shown that the novel disaccharide LacdiNAc group is detected in glycoproteins derived from a variety of human tumors and cancer cells, and its expression is closely associated with the tumor progression or regression. The expression of the disaccharide group and that of *β*4GalNAcT3 and *β*4GalNAcT4 appear to be regulated in a tissue-specific manner. The tissue-specific expression of the LacdiNAc group strongly indicates that the disaccharide group becomes a potent diagnostic marker for particular human cancers. Further studies are required to elucidate the biological significance of the LacdiNAc group in human tumors and cancer cells.

## Figures and Tables

**Table 1:** Expression of LacdiNAc group in human tumors and cancer cells.

Types of tumors/cells	LacdiNAc	*β*4GalNAcT	References
Breast cancer	↓	ND	[19]
Neuroblastoma	ND	*β*4GalNAcT3 ↓	[28]
Colon cancer	ND	*β*4GalNAcT3 ↑	[38]
Prostate cancer	(PSA)↑	*β*4GalNAcT4 ↑	[20, 21, 33]
Ovarian cancer/SKOV3	↑	ND	[22]
Pancreatic cancer/Capan-1	(RNase I)↑	ND	[23]

ND: not determined.

Arrows ↑ and ↓ indicate an increase and a decrease of the expression, respectively.

## References

[1] van Die I, Cummings RD (2010). Glycan gimmickry by parasitic helminths: a strategy for modulating the host immune response?. *Glycobiology*.

[2] Manzella SM, Hooper LV, Baenziger JU (1996). Oligosaccharides containing *β*1, 4-linked N-acetylgalactosamine, a paradigm for protein-specific glycosylation. *Journal of Biological Chemistry*.

[3] Furukawa K, Kitamura N, Sato T, Hiraizumi S (2001). Differentiation-associated expression of *β*-N-acetyl-galactosaminylated N-linked oligo-saccharides in mammary epithelial cells. *Advances in Experimental Medicine and Biology*.

[4] Sakiyama T, Kabayama M, Tomita M (1998). Distribution of glycoproteins with *β*-N-acetylgalactosaminylated N-linked sugar chains among bovine tissues. *Biochimica et Biophysica Acta*.

[5] Green ED, van Halbeek H, Boime I, Baenziger JU (1985). Structural elucidation of the disulfated oligosaccharide from bovine lutropin. *Journal of Biological Chemistry*.

[6] Green ED, Baenziger JU (1988). Asparagine-linked oligosaccharides on lutropin, follitropin, and thyrotropin. I. Structural elucidation of the sulfated and sialylated oligosaccharides on bovine, ovine, and human pituitary glycoprotein hormones. *Journal of Biological Chemistry*.

[7] Green ED, Baenziger JU (1988). Asparagine-linked oligosaccharides on lutropin, follitropin, and thyrotropin. II. Distributions of sulfated and sialylated oligosaccharides on bovine, ovine, and human pituitary glycoprotein hormones. *Journal of Biological Chemistry*.

[8] Yan SB, Chao YB, van Halbeek H (1993). Novel Asn-linked oligosaccharides terminating in GalNAc*β*(1→4)[Fuc*α*(1→3)]GlcNAc*β*(1 → *·*) are present in recombinant human protein C expressed in human kidney 293 cells. *Glycobiology*.

[9] Nakata N, Furukawa K, Greenwalt DE, Sato T, Kobata A (1993). Structural study of the sugar chains of CD36 purified from bovine mammary epithelial cells: occurrence of novel hybrid-type sugar chains containing the Neu5Ac*α*2→6GalNAc*β*1→4GlcNAc and the Man*α*1→2Man*α*1→3Man*α*1→6Man groups. *Biochemistry*.

[10] Sato T, Takio K, Kobata A, Greenwalt DE, Furukawa K (1995). Site-specific glycosylation of bovine butyrophilin. *Journal of Biochemistry*.

[11] Dell A, Morris HR, Easton RL (1995). Structural analysis of the oligosaccharides derived from glycodelin, a human glycoprotein with potent immunosuppressive and contraceptive activities. *Journal of Biological Chemistry*.

[12] Siciliano RA, Morris HR, Bennett HPJ, Dell A (1994). O-glycosylation mimics N-glycosylation in the 16-kDa fragment of bovine pro-opiomelanocortin: the major O-glycan attached to Thr-45 carries SO_4_-4GalNAc*β*1-4GlcNAc*β*1-, which is the archetypal non-reducing epitope in the N-glycans of pituitary glycohormones. *Journal of Biological Chemistry*.

[13] Dell A, Chalabi S, Easton RL (2003). Murine and human zona pellucida 3 derived from mouse eggs express identical O-glycans. *Proceedings of the National Academy of Sciences of the United States of America*.

[14] Fiete D, Srivastava V, Hindsgual O, Baenziger JU (1991). A hepatic reticuloendothelial cell receptor specific for SO_4_-4GalNAc-*β*1, 4GlcNAc*β*1, 2Man*α* that mediates rapid clearance of lutropin. *Cell*.

[15] Baenziger JU, Kumar S, Brodbeck RM, Smith PL, Beranek MC (1992). Circulatory half-life but not interaction with the lutropin/chorionic gonadotropin receptor is modulated by sulfation of bovine lutropin oligosaccharides. *Proceedings of the National Academy of Sciences of the United States of America*.

[16] Roseman DS, Baenziger JU (2000). Molecular basis of lutropin recognition by the mannose/GalNAc-4-S0_4_ receptor. *Proceedings of the National Academy of Sciences of the United States of America*.

[17] Ujita M, Furukawa K, Aoki N (1993). A change in soybean agglutinin binding patterns of bovine milk fat globule membrane glycoproteins during early lactation. *FEBS Letters*.

[18] Sato T, Taka J, Aoki N, Matsuda T, Furukawa K (1997). Expression of *β*-N-acetylgalactosaminylated N-linked sugar chains is associated with functional differentiation of bovine mammary gland. *Journal of Biochemistry*.

[19] Kitamura N, Guo S, Sato T (2003). Prognostic significance of reduced expression of *β*-N-acetylgalactosaminylated N-linked oligosaccharides in human breast cancer. *International Journal of Cancer*.

[20] Hirano K, Nakamura T, Amano J (2009). Method for determining prostate cancer. *International Patent*.

[21] Fukushima K, Satoh T, Baba S, Yamashita K (2010). *α*1,2-fucosylated and *β*-N-acetylgalactosaminylated prostate-specific antigen as an efficient marker of prostatic cancer. *Glycobiology*.

[22] MacHado E, Kandzia S, Carilho R, Altevogt P, Conradt HS, Costa J (2011). N-glycosylation of total cellular glycoproteins from the human ovarian carcinoma SKOV3 cell line and of recombinantly expressed human erythropoietin. *Glycobiology*.

[23] Peracaula R, Royle L, Tabarés G (2003). Glycosylation of human pancreatic ribonuclease: differences between normal and tumor states. *Glycobiology*.

[24] Hakomori S (1981). Glycosphingolipids in cellular interaction, differentiation, and oncogenesis. *Annual Review of Biochemistry*.

[25] Hakomori S (1996). Tumor malignancy defined by aberrant glycosylation and sphingo(glyco)lipid metabolism. *Cancer Research*.

[26] Heimburg-Molinaro J, Lum M, Vijay G, Jain M, Almogren A, Rittenhouse-Olson K (2011). Cancer vaccines and carbohydrate epitopes. *Vaccine*.

[27] Sato T, Furukawa K, Greenwalt DE, Kobata A (1993). Most bovine milk fat globule membrane glycoproteins contain asparagine-linked sugar chains with GalNAc*β*1→4GlcNAc groups. *The Journal of Biochemistry*.

[28] Hsu W-M, Che M-I, Liao Y-F (2011). B4GALNT3 expression predicts a favorable prognosis and suppresses cell migration and invasion via *β*1 integrin signaling in neuroblastoma. *The American Journal of Pathology*.

[29] Ohyama C, Hosono M, Nitta K (2004). Carbohydrate structure and differential binding of prostate specific antigen to *Maackia amurensis lectin* between prostate cancer and benign prostate hypertrophy. *Glycobiology*.

[30] Tabarés G, Radcliffe CM, Barrabés S (2006). Different glycan structures in prostate-specific antigen from prostate cancer sera in relation to seminal plasma PSA. *Glycobiology*.

[31] Tajiri M, Ohyama C, Wada Y (2008). Oligosaccharide profiles of the prostate specific antigen in free and complexed forms from the prostate cancer patient serum and in seminal plasma: a glycopeptide approach. *Glycobiology*.

[32] Okada T, Sato Y, Kobayashi N (2001). Structural characteristics of the N-glycans of two isoforms of prostate-specific antigens purified from human seminal fluid. *Biochimica et Biophysica Acta*.

[33] Peracaula R, Tabarés G, Royle L (2003). Altered glycosylation pattern allows the distinction between prostate-specific antigen (PSA) from normal and tumor origins. *Glycobiology*.

[34] Yamashita K, Ohkura T, Umetsu K, Suzuki T (1992). Purification and characterization of a Fuc*α*1→2Gal*β*1→ and GalNAc*β*1→ specific lectin in root tubers of *Trichosanthes japonica*. *Journal of Biological Chemistry*.

[35] Sato T, Gotoh M, Kiyohara K (2003). Molecular cloning and characterization of a novel human *β*1,4-N-Acetylgalactosaminyltransferase, *β*4GalNAc-T3, responsible for thesynthesis of N,N′-diacetyllactosediamine, GalNAc*β*1-4GlcNAc. *Journal of Biological Chemistry*.

[36] Gotoh M, Sato T, Kiyohara K (2004). Molecular cloning and characterization of *β*1,4-N-acetyl-galactosaminyltransferases IV synthesizing N,N′-diacetyllactosedi-amine. *FEBS Letters*.

[37] Ikehara Y, Sato T, Niwa T (2006). Apical Golgi localization of N,N′-diacetyllactosediamine synthase, *β*4GalNAc-T3, is responsible for LacdiNAc expression on gastric mucosa. *Glycobiology*.

[38] Huang J, Liang J-T, Huang H-C (2007). *β*1,4-N-acetylgalactosaminyltransferase III enhances malignant phenotypes of colon cancer cells. *Molecular Cancer Research*.

[39] Smith PL, Baenziger JU (1992). Molecular basis of recognition by the glycoprotein hormone-specific N-acetylgalactosamine-transferase. *Proceedings of the National Academy of Sciences of the United States of America*.

[40] Mengeling BJ, Manzella SM, Baenziger JU (1995). A cluster of basic amino acids within an *α*-helix is essential for *α*-subunit recognition by the glycoprotein hormone N-acetylgalactosaminyltransferase. *Proceedings of the National Academy of Sciences of the United States of America*.

[41] Miller E, Fiete D, Blake NMJ (2008). A necessary and sufficient determinant for protein-selective glycosylation *in vivo*. *Journal of Biological Chemistry*.

[42] Fiete D, Beranek M, Baenziger JU (2012). Peptide-specific transfer of *N*-acetylgalactosamine to *O*-linked glycans by the glycosyltransferases *β*1,4-N-acetylgalactosaminyl transferase 3 (*β*4GalNAc-T3) and *β*4GalNAc-T4. *Journal of Biological Chemistry*.

[43] Fiete D, Beranek M, Baenziger JU (2012). Molecular basis for protein-specific transfer of N-acetyl-galactosamine to N-linked glycans by the glycosyltransferases *β*1,4-N-acetylgalactosaminyl transferase 3 (*β*4GalNAc-T3) and *β*4GalNAc-T4. *Journal of Biological Chemistry*.

[44] Dharmesh SM, Skelton TP, Baenziger JU (1993). Co-ordinate and restricted expression of the ProXaaArg/Lys-specific GalNAc-transferase and the GalNAc*β*1,4GlcNAc*β*1,2Man*α*-4-sulfotransferase. *Journal of Biological Chemistry*.

[45] van den Nieuwenhof IM, Schiphorst WECM, van Die I, van den Eijnden DH (1999). Bovine mammary gland UDP-GalNAc:GlcNAc*β*-R *β*1→4-N-acetylgalactosaminyltransferase is glycoprotein hormone nonspecific and shows interaction with *α*-lactalbumin. *Glycobiology*.

[46] Do K-Y, Do S-I, Cummings RD (1995). *α*-Lactalbumin induces bovine milk *β*1,4-galactosyltransferase to utilize UDP-GalNAc. *Journal of Biological Chemistry*.

[47] Kido M, Sakiyama T, Ichinose M, Miki K, Furukawa K (2000). Occurrence of *β*-1,4-N-acetylgalactosaminylated N-linked oligosaccharides in *β*-1,4-galactosyltransferase I-knockout mouse. *Research Communications in Biochemistry and Cell and Molecular Biology*.

[48] Park EI, Manzella SM, Baenziger JU (2003). Rapid clearance of sialylated glycoproteins by the asialoglycoprotein receptor. *Journal of Biological Chemistry*.

[49] Park EI, Mi Y, Unverzagt C, Gabius H-J, Baenziger JU (2005). The asialoglycoprotein receptor clears glycoconjugates terminating with sialic acid *α*2,6GalNAc. *Proceedings of the National Academy of Sciences of the United States of America*.

[50] Breloy I, Pacharra S, Ottis P, Bonar D, Grahn A, Hanisch FG (2012). O-linked N′,N′-diacetyllactosamine (LacdiNAc)-modified glycans in extracellular matrix glycoproteins are specifically phosphorylated at subterminal N-acetylglucosamine. *Journal of Biological Chemistry*.

